# Postpartal recurrent non-ST elevation myocardial infarction in essential thrombocythaemia: case report and review of the literature

**DOI:** 10.1186/1477-9560-8-12

**Published:** 2010-06-17

**Authors:** Spyridon Arampatzis, Ioannis Stefanidis, Vassilios Lakiopoulos, Luigi Raio, Daniel Surbek, Markus G Mohaupt

**Affiliations:** 1 Department of Nephrology/Hypertension, University of Bern, Berne, Switzerland; 2 Department of Nephrology, University of Thessaly, Larissa, Greece; 3 Women's Hospital, University of Bern, Berne, Switzerland

## Abstract

Normal pregnancy corresponds to a procoagulant state. Acute myocardial infarction during pregnancy is rare, yet considering the low non-pregnant risk score of childbearing women it is still surprisingly frequent. We report a case of postpartum recurrent non-ST elevation myocardial infarction in a 40-year-old caucasian woman with essential thrombocythaemia in the presence of a positive JAK-2 mutation and an elevated anti-cardiolipin IgM antibody titer. In the majority of cases of myocardial infarction in pregnancy or in the peripartal period, atherosclerosis, a thrombus or coronary artery dissection is observed. The combination of essential thrombocythaemia and elevated anti-cardiolipin IgM antibody titer in the presence of several cardiovascular risk factors seems to be causative in our case. In conclusion, with the continuing trend of childbearing at older ages, rare or unlikely conditions leading to severe events such as myocardial infarction must be considered in pregnant women.

## Introduction

Essential thrombocythaemia (ET) is a chronic myeloproliferative disorder characterized by a sustained elevated platelet count with a tendency to both thrombosis and hemorrhage [1,2]. In ET the median age of presentation is 60 years with female predominance [3] and has a favorable outcome [4]. A small subset of patients is being diagnosed at an earlier age [5,6]. Young women with ET constitute a special group due to their anticipated long survival and childbearing potential [7-9]. Pregnancies in ET patients are likely to be complicated, primarily due to first trimester spontaneous abortions but for those carried to term, obstetric or thrombohemorrhagic complications are rare [10].

Pregnancy is an acquired risk factor for thromboembolism associated with increased coagulation and decreased fibrinolysis [11]. Hemodynamic and hormonal alterations during pregnancy may further potentiate the risk of vascular events [12,13]. Although rare, acute myocardial infarction does complicate pregnancy and is estimated to occur in about 6 per 100,000 women during the peripartal period [14].

Pregnancy-related complications in patients with ET remains a challenge as platelet count has not been shown to represent a risk factor for pregnancy complications, nor the use of aspirin has been demonstrated to influence pregnancy outcome [9]. We describe a case involving recurrent non-ST elevation myocardial infarction in the immediate postpartum period in a young woman with ET. We review the current literature for pregnancy-related risk factors of myocardial infarction with respect to ET.

## Case report

A 40-year-old caucasian woman, gravida 3 para 2, developed postpartal arterial hypertension. Her first pregnancy, three years earlier, had been complicated by an early spontaneous abortion at gestational week 7. In the following pregnancy, one year later, while on prophylactic low-molecular weight heparin due to the previous miscarriage, a cesarean section was performed at gestational week 32 due to intrauterine fetal growth restriction (IUGR), infant birthweight 810 g, 1-, 5-, and 10-min Apgar scores of 7, 9 and 10, respectively; umbilical cord arterial blood pH: 7.00. During the present pregnancy, low-dose aspirin was given from gestational week 13 to week 37 due to IUGR in the former pregnancy. Shortly after an uneventful elective cesarean section performed in the 39^th ^week (infant birthweight 2520 g, 1-, 5-, and 10-min Apgar scores of 9, 9 and 10, respectively; umbilical cord arterial blood pH: 7.29) monotherapy with 50 mg metoprolol was started due to postpartal hypertension.

The patient had the same partner since the first pregnancy and no history of spontaneous bleeding, thrombosis nor had she been diagnosed to have elevated platelet counts requiring treatment. During the present pregnancy platelet counts were initially elevated but continuously decreased from 598 G/L to 346 G/L at the time of caesarean delivery.

Risk factors for coronary heart or thromboembolic diseases, including smoking, hyperlipidemia, diabetes mellitus or atrial fibrillation were absent except for a positive family history of coronary artery disease and overweight (body mass index: 28.6 kg/m^2^). No medication or illicit drugs were taken. The patient was breastfeeding.

Three weeks after delivery, she complained of shortness of breath and acute retrosternal pain accompanied by severe migraine and arterial hypertension. Despite 10 mg nifedipin intake, hypertension and retrosternal pain persisted and the patient was referred to a cardiologist with a supine blood pressure of 180/85 mm Hg and a regular heart rate at 90 bpm. The clinical examination was otherwise normal. Her initial ECG revealed a myocardial injury pattern compatible with a non-significant ST-elevation of the inferior leads.

Laboratory studies (Table 1) showed an elevated platelet count (708 G/L) and increased troponin-T levels. A chest x-ray and a spiral computer tomography angiography scan rule out pulmonary thromboembolism. Given the severe recurrent migraine episodes, a stroke was excluded by a cerebral computer tomography scan.

**Table 1:** Laboratory parameters at acute coronary syndromes (ACS) episodes

	1^st ^episode ACS	2^nd ^episode ACS	Values	Normal range
Hb	137	139	g/L	121-154

Hct	40	42	%	36-44

Lc	8.6	8.0	G/L	3.5-10.5

Tc	**708**	**559**	G/L	140-380

				

CK max	**280**	**196**	U/L	<170

Troponin T max	**0.620**	**0.354**	μg/L	<0.010

				

**Antibodies**				

ANA		1:160		<1:80

Anti-dsDNA-		32		<200

anti-histone		0.3		<1

IgM anticardiolipin		**13.1**	MPL-U/mL	<5

IgG anticardiolipin		3	GPL-U/mL	<10

**Figure 1:**
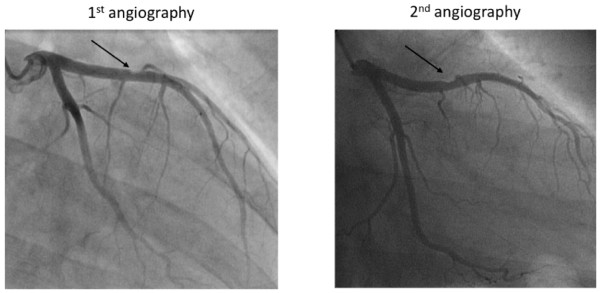
**The LAD lesion visible at the first and the second coronary angiography are depicted on the left and right hand side, respectively, and indicated by arrows**.

During the night, creatinine kinase rose as did troponin-T (187 to 280 U/l and 0.129 to 0.319 ng/ml, respectively). With the clinical and laboratory findings suggesting anterior wall myocardial infarction, the patient was started on aspirin, intravenous heparin and nitroglycerin, and a coronary angiography was performed (Fig. 1). This revealed a non-significant plaque in the proximal left anterior descending (LAD) area of an otherwise normal coronary artery tree. She was started on amlodipin, metoprolol, simvastatin, aspirin, and clopidogrel, and the chest pain resolved. The further in-hospital course was uneventful and 7 days after the coronary angiography the patient was discharged on aspirin, low molecular weight heparin, a statin, metoprolol and amlodipin, with an appointment, 5 weeks later, for a further hematology workup due to the elevated platelet count and potential additive procoagulatory risk factors.

After 4 weeks the patient was readmitted to the emergency unit complaining of severe chest pain radiating to the neck. She reported having stopped aspirin as well as metoprolol and amlodipin 10 days earlier. The clinical examination revealed a blood pressure of 150/90 mmHg and a heart rate of 62 bpm. The ECG displayed a regular rhythm, yet inferior lead non-significant ST-segment elevations. The troponin-T levels were elevated (Table 1) and echocardiography confirmed a slight anterior wall hypokinesia already observed during the first ischemic episode. Medication was restarted and the second coronary angiography showed a hemodynamically non-relevant plaque (10-30%) within the distal branch of the LAD, with no further new findings (Fig. 1). At this time, paradoxical embolism and myocardial inflammatory disease was ruled out by transesophageal echocardiography and by cardiac nuclear magnetic resonance scan, respectively.

Further hematological work-up confirmed the diagnosis of ET by a persistent elevated platelet count, a positive JAK-2 (V617F)-mutation, a matching bone marrow finding without evidence for iron deficiency or infection. Potential hereditary risk factors for thrombophilia (factor V Leiden, prothrombin gene mutations, methylenetetrahydrofolate reductase (MTHFR), factor XIII, and PAI-1) were excluded. Cytogenetics for BCR-ABL, t(9;22), were negative. IgM anticardiolipin antibodies were transiently elevated and ANA was 1:160 with negative anti-dsDNA and anti-Histone antibodies.

Oral hydroxyurea was added to aspirin, but had to be discontinued due to severe alopecia. In response to alternate treatment with low dose peg-interferon α-2a, platelet count normalized. At the one-year follow-up, the patient presented with normal blood pressure and remained in remission for hematological and cardiac disease while remaining on aspirin.

## Discussion

Pregnancy is not commonly considered a risk factor for acute myocardial infarction, however pregnancy increase the risk of acute myocardial infarction 3- to 4-fold [14,15]. Many risk factors are unique for pregnancy-related acute myocardial infarction and several diagnostic steps are often required for the myocardial infarction confirmation (Appendix 1).

Cardiac function and hormonal milieu are unfavorably altered in pregnancy whereas cardiac output is increased in the presence of elevated levels of estrogen and progesterone [12,13]. The hypercoagulable state of pregnancy [11], in the presence of increased vascular reactivity [16], may further magnify the risk of myocardial infarction. Progesterone excess and postpartal degeneration of the matrix in the medial and intimal sections of the coronary arteries may contribute to flow alterations and artery dissections [17].

Superimposed hypertension, as in the reported case may further damage blood vessels already weakened by hemodynamic stress and hormonal alterations. Overall, an increasing prevalence of cardiovascular risk factors with advanced maternal age contributes to pregnancy-associated complications [18,19]. More than 70% of patients with ET and recurrent thrombosis have multiple cardiovascular risk factors [20,21]. Overweight, arterial hypertension and a positive family history were present in this case, yet all were moderate and hypertension only recently developed.

In a recent retrospective review of 228 reported cases of pregnancy-related acute myocardial infarction, morphology of the coronary arteries was present in 164 cases. Atherosclerosis with or without intracoronary thrombus was found in 70 cases (43%), and definite or probable coronary thrombus without evidence of atherosclerotic disease was present in 22 (13%). Coronary artery dissection was verified in 24%, spasms and embolus in 2% and 1% respectively. Normal coronary arteries were found in 20% [16,22]

Published studies on ET pregnancies report live birth rates of 50-70% and spontaneous abortion rates of 25-50% [23,24]. In a recent report of 103 pregnancies that occurred in 62 women with ET, about 50% of first pregnancies experienced complications, although no case of acute coronary syndrome (ACS) or myocardial infarction was reported during pregnancy or postpartum [9].

Despite the fact that a decrease in platelet count during pregnancies is well documented, pregnancies in ET patients frequently end in early spontaneous abortions, during the first trimester [25]. Their occurrence cannot be predicted from the disease course, platelet count, or a specific therapy. The use of aspirin did not improve pregnancy outcome in a study of 34 patients with ET by Tefferi and coworkers [7,10]. In addition control of the platelet count alone should not be taken as an appropriate surrogate end point to judge the efficacy of a treatment for ET [26]. In a randomized study comparing anagrelide vs. hydroxyurea therapy (plus low-dose aspirin in both groups) in ET [23], an excess of vascular events was found in the anagrelide group despite a reduction in the platelet count similar to that in the hydroxyurea group. If the platelet count decreases insufficiently in patients with ET despite pregnancy, interferon is considered the agent of choice, omitting the teratogenicity of cytoreductive agents [7,27].

Our patient's history of previous early spontaneous abortion, IUGR and the presence of anticardiolipin antibodies suggest the possibility of an incomplete antiphospholipid antibody syndrome, which represent the most common acquired thrombophilia of pregnancy and has been associated with myocardial infarction [28,29].

Antiphospholipid antibody syndromes may also be associated with autoimmune diseases such as systemic lupus erythematosus, which can cause pericarditis and myocarditis [30]. In the present case, autoantibody screening for ANA was borderline and anti-dsDNA and anti-Histone antibody testing were negative. A nuclear magnetic resonance examination of the heart demonstrated a normal myocardium with no signs of inflammation.

Our patient had experienced a severe migraine attack which has also been found to be a risk factor for myocardial infarction during pregnancy [14]. The possible underlying mechanism may be a generalized vasospasm that makes coronary arteries susceptible to spasms [31].

Despite the fact that ET-pregnancies carried to term are rarely complicated by thrombohemorrhagic events our patient had experienced recurrent postpartal ACS in the presence of essential thrombocytosis and elevated antio-cardiolipin IgM antibodies. Since patients with ET seems to have an increased prevalence of antiphospholipid antibodies which may be associated with thrombosis [32] it is not surprising that both episodes of ACS occurred after aspirin discontinuation. We can only speculate that the conjunction of primary etiological factors such as ET and the transiently elevated antiphospholipid antibody titer in the presence of several cardiovascular risk factors (advanced maternal age, hypertension, postpartal vascular changes, coronary plaque) and the lack of antiplatelet therapy finally contributed to recurrent myocardial ischemia.

There are several recommendations [33] that women whose pregnancies are characterized by fetal complications, such as unexplained fetal growth retardation and stillbirth, should be tested for genetic or acquired markers of thrombophilia as well as antiphospholipid antibodies and autoimmune disease. We propose that, with the continuing trend of childbearing at older ages, maternal complications like the ones described here should be added to the conditions requiring a similar work-up to allow for closer monitoring, or even prophylactic therapy during further pregnancies and beyond.

## List of abbreviations

ET: essential thrombocythaemia; AMI: acute myocardial infarction; ACS: acute coranary syndrome; LAD: left anterior descending; MTHFR: methylenetetrahydrofolate reductase; CK: creatine kinase.

## Consent

Written informed consent was obtained from the patient for publication of this case report and accompanying images. A copy of the written consent is available for review by the Editor-in-Chief of this journal.

## Competing interests

The authors declare that they have no competing interests.

## Authors' contributions

SA, MGM, RL, DS, made substantial contributions to patient care and to the preparation of the manuscript. IS and VL contributed to the manuscript preparation. All authors read and approved the final manuscript.

## Appendix

Appendix 1.

Diagnostic approach of suspected acute myocardial infarction (AMI) in pregnant women

### AMI typical clinical symptoms

Symptoms mimicking myocardial ischemia may also be present during healthy pregnancies

### Electrocardiographic changes

ST-segment depression may also be present due induction of anesthesia for cesarean section and may persist after elective cesarean section

### Cardiac markers

Troponin I levels: is the most sensitive marker for AMI. The majority of healthy pregnant women remain below the upper limit of normal Troponin I levels after delivery.

Creatine kinase/CK-MB: can be significantly elevated up to 24 h after delivery in healthy pregnant women

### Echocardiogram/Stress echocardiography

Safe and accurate for evaluating wall-motion abnormalities

### Exercise testing

Submaximal protocol evaluation (70% of maximal predicted heart rate) with fetal monitoring for the diagnosis of myocardial ischemia or risk stratification following AMI

### Radionuclide imaging and cardiac MRI

Both modalities may add further information with overall small or none fetal exposure to radiation

### Cardiac catheterization

Possible increased risk of coronary dissection and great risk of fetal exposure to radiation

Both the cardiologist and obstetrician should establish the treatment plan
